# Circular RNA hsa_circ_0000190 Facilitates the Tumorigenesis and Immune Evasion by Upregulating the Expression of Soluble PD-L1 in Non-Small-Cell Lung Cancer

**DOI:** 10.3390/ijms23010064

**Published:** 2021-12-22

**Authors:** Yung-Hung Luo, Yi-Ping Yang, Chian-Shiu Chien, Aliaksandr A. Yarmishyn, Afeez Adekunle Ishola, Yueh Chien, Yuh-Min Chen, Ping-Hsing Tsai, Tzu-Wei Lin, Mong-Lien Wang, Shih-Hwa Chiou

**Affiliations:** 1 Department of Chest Medicine, Taipei Veterans General Hospital, Taipei 11217, Taiwan; hecterlo@gmail.com (Y.-H.L.); ymchen@vghtpe.gov.tw (Y.-M.C.); 2 School of Medicine, National Yang Ming Chiao Tung University, Taipei 11221, Taiwan; ypyang3@vghtpe.gov.tw (Y.-P.Y.); monglien@gmail.com (M.-L.W.); 3 Institute of Clinical Medicine, National Yang Ming Chiao Tung University, Taipei 11221, Taiwan; 4 Department of Medical Research, Taipei Veterans General Hospital, Taipei 11217, Taiwan; polo661124@yahoo.com.tw (C.-S.C.); yarmishyn@gmail.com (A.A.Y.); aaishola01@gmail.com (A.A.I.); g39005005@gmail.com (Y.C.); figatsai@gmail.com (P.-H.T.); backyard0826@gmail.com (T.-W.L.); 5 School of Pharmaceutical Sciences, National Yang Ming Chiao Tung University, Taipei 11221, Taiwan; 6 Institute of Pharmacology, National Yang Ming Chiao Tung University, Taipei 11221, Taiwan; 7 Taiwan International Graduate Program in Molecular Medicine, National Yang Ming Chiao Tung University and Academia Sinica, Taipei 11221, Taiwan; 8 Institute of Food Safety and Health Risk Assessment, National Yang Ming Chiao Tung University, Taipei 11221, Taiwan

**Keywords:** lung cancer, circular RNA, immune checkpoints, immune evasion, programmed death-ligand 1

## Abstract

Lung cancer is the leading cause of death from cancer in Taiwan and throughout the world. Immunotherapy has revealed promising and significant efficacy in NSCLC, through immune checkpoint inhibition by blocking programmed cell death protein (PD)-1/PD-1 ligand (PD-L1) signaling pathway to restore patients’ T-cell immunity. One novel type of long, non-coding RNAs, circular RNAs (circRNAs), are endogenous, stable, and widely expressed in tissues, saliva, blood, urine, and exosomes. Our previous results revealed that the plasma level of hsa_circ_0000190 can be monitored by liquid-biopsy-based droplet digital PCR and may serve as a valuable blood-based biomarker to monitor the disease progression and the efficacy of immunotherapy. In this study, hsa_circ_0000190 was shown to increase the PD-L1 mRNA-mediated soluble PD-L1 (sPD-L1) expression, consequently interfering with the efficacy of anti-PD-L1 antibody and T-cell activation, which may result in immunotherapy resistance and poor outcome. Our results unraveled that hsa_circ_0000190 facilitated the tumorigenesis and immune evasion of NSCLC by upregulating sPD-L1 expression, potentially developing a different aspect in elucidating the molecular immunopathogenesis of NSCLC. Hsa_circ_0000190 upregulation can be an effective indicator for the progression of NSCLC, and hsa_circ_0000190 downregulation may possess a potential therapeutic value for the treatment of NSCLC in combination with immunotherapy.

## 1. Introduction

Lung cancer (LC) is the leading cause of death worldwide, and more than 85% of LC cases belong to non-small-cell lung carcinoma (NSCLC) [1]. Most LC patients have locally advanced disease or distant metastases at the time of diagnosis. In the past decade, the emergence of targeted therapies and immunotherapies has led to remarkable improvement in the treatment efficacy for NSCLC, but the survival rate for advanced NSCLC still remains dismal [1,2,3,4,5,6,7,8]. Despite the significant advancement in the medical treatment of LC, fewer than 10% of advanced NSCLC patients survive for more than five years. Hence, the development of superior diagnostic and therapeutic approaches is critical for a better understanding of the molecular pathogenesis of LC. In turn, such advancements can contribute to the discovery of novel biomarkers for cancer detection and molecular treatment targets for LC, as well as more personalized treatment of LC patients.

Circular RNAs (circRNAs) are non-coding RNAs that comprise a circular loop with multiple microRNA (miRNA) binding sites and function as miRNA sponges, to compete with endogenous miRNAs and, subsequently, regulate gene expression [9,10].

Owning to the closed structure, circRNAs are relatively stable and particularly resistant to RNA-degrading endonucleases. Hence, circRNAs demonstrate a much longer circulatory half-life, as compared with linear RNAs, implicating the potential as promising cancer biomarkers [10]. Many circRNAs in the cytoplasm function in the circRNA–miRNA–mRNA axis and are involved in proliferation and survival pathways in different kinds of cancer [11]. Our previous research demonstrated that the levels of hsa_circ_0000190 and hsa_circ_0001649 increased in lung cancer cells and patients’ plasma. Additionally, the level of hsa_circ_0000190 was related to clinicopathological characteristics and the treatment response of patients with lung cancer [12]. Therefore, hsa_circ_0000190 might be a valuable blood-based biomarker to evaluate the prognosis of LC and the efficacy of immunotherapy by ddPCR-based liquid biopsy. 

Multiple studies have reported the role of circRNAs in regulating the expression of PD-L1 through circRNA–miRNA–mRNA axis. A number of circRNAs, such as circBART2.2, hsa_circ_0136666, circ-keratin 6c, CDR1-AS, circ-VIM, hsa_circ_0003288, CircCHST15, circ-CPA4, hsa-circRNA-002178, and Circ_0000284 were shown to affect PD-L1 expression, leading to tumor immune escape in various types of cancers, including nasopharyngeal carcinoma, colorectal cancer, esophageal cancer, hepatocellular carcinomas (HCC), and lung cancer [13,14,15,16,17,18,19,20,21,22]. Our previous study discovered the correlation between hsa_circ_0000190 plasma level and PD-L1 level in patients with lung cancer. Upregulated plasma hsa_circ_0000190 level was associated with poor response to immunotherapy [12]. Substantial evidence suggested that circRNAs play essential roles in the tumor immune microenvironment (TIME) and regulate immune checkpoint genes to affect the therapeutic efficacy of immunotherapy. Further research regarding the effect of circRNAs on antitumor immunity and the treatment response of immunotherapy will benefit the management of advanced lung cancer [23].

In this study, the influence of the circRNA–miRNA–mRNA axis on the immune system and immune checkpoints is evaluated, to elucidate the potential regulatory pathway of the lung cancer immune evasion process. Additionally, the combined effects of immune checkpoint inhibitors and circRNAs inhibitors are investigated, in order to develop novel strategies against the immune evasion of lung cancer cells and to strengthen the efficacy of lung cancer immunotherapy.

## 2. Results

### 2.1. Overexpression of hsa_circ_0000190 Promoted Tumorigenesis of NSCLC In Vitro

**Figure 1:**
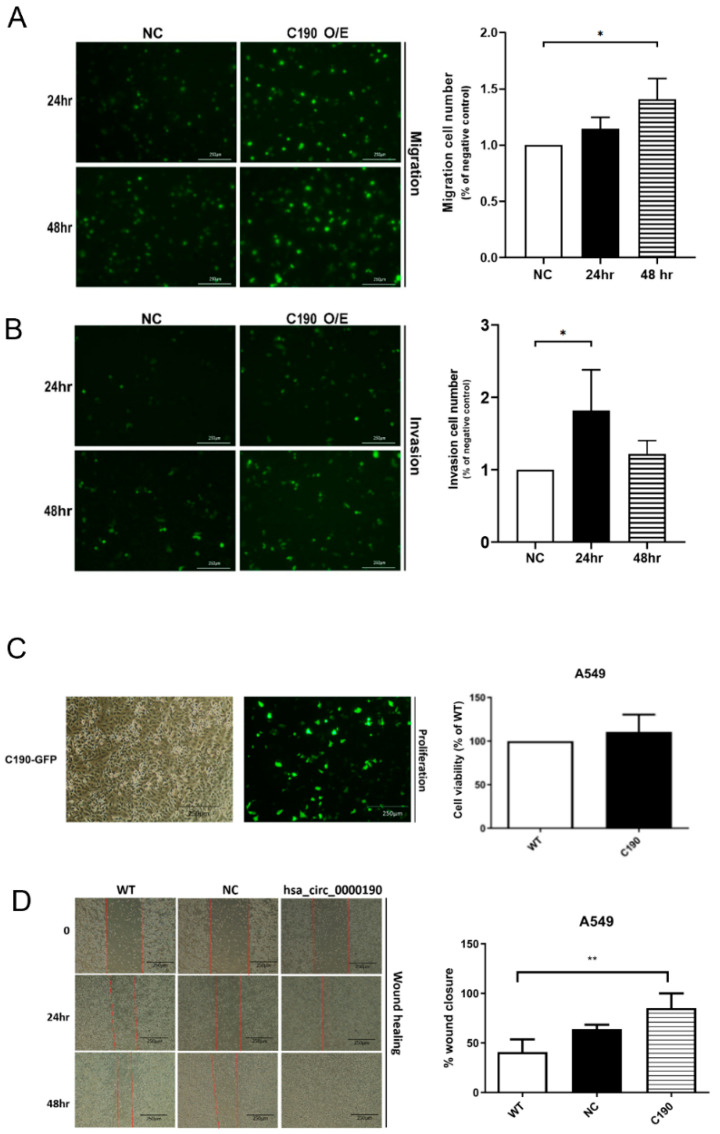
Overexpression (O/E) of hsa_circ_0000190 (C190) promoted tumorigenesis of NSCLC: (**A**) A549 with overexpression of C190 demonstrated the significantly increased cell migration and (**B**) invasion compared with negative control (NC) cells (* *p* < 0.05); (**C**) A549 with overexpression of C190 demonstrated the tendency of increased proliferation; (**D**) A549 cells with overexpression of C190 showed increased ability of wound healing (** *p* < 0.01). All experiments were performed independently in triplicate.

To analyze the biological role of C190 in NSCLC, overexpression vectors targeting C190 were delivered into NSCLC cell lines. In Figure 1A,B, the migration and invasion of A549 cells with overexpression of C190 through 8-μM pore in transwells is shown. Migration and invasion cells at the transwell bottom were stained with GFP and observed under a microscope (magnification ×100). The average number of cells in the three light-field microscopies is shown. The data show that A549 with overexpression of C190 demonstrated significantly increased cell migration and invasion, compared with the negative control (NC) cells (* *p* < 0.05). 

In Figure 1C, the proliferation of A549 cells with overexpression of C190 is shown. Briefly, 5000 cells were dispensed into each well of a 96-well plate. Cells were pre-incubated in the incubator for 24 h. After 24 h, 10 µL of CCK-8 solution was added to each well and incubated for 4 h. A549 with overexpression of C190 demonstrated the tendency of increased proliferation.

In Figure 1D, wound-healing analysis of A549 with overexpression of C190 is demonstrated by one field of view at each time point. Evidence of cell migration is seen post-wound creation and the cell-free space steadily decreases over time as the cells move to close the space completely. A549 cells with overexpression of C190 demonstrated an increased ability of wound healing. 

### 2.2. Knockdown of hsa_circ_0000190 Hindered Tumorigenesis of NSCLC In Vitro

**Figure 2:**
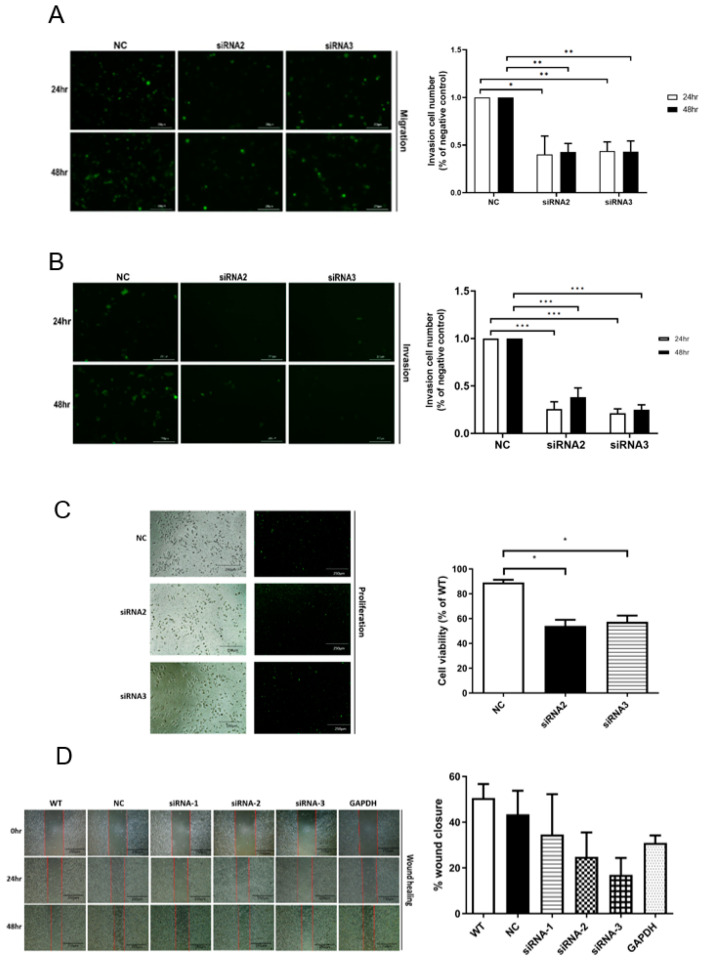
Knockdown of hsa_circ_0000190 hindered tumorigenesis of NSCLC in vitro: (**A**) A549 cells with knockdown of C190 by siRNA2 and siRNA3 showed significantly decreased migration ability, compared with NC (* *p* < 0.05, ** *p* < 0.01); (**B**) A549 cells with knockdown of C190 showed significantly decreased invasion ability, compared with NC (*** *p* < 0.001); (**C**) A549 cells with knockdown of C190 showed significantly decreased proliferation ability, compared with NC; (**D**) A549 cells with knockdown of C190 showed a tendency toward decreased wound-healing ability, compared with the NC. All experiments were performed independently in triplicate.

To analyze the influence of C190 on tumorigenesis of NSCLC, siRNA targeting C190 was used for its expression knockdown. The specific siRNAs (siRNA2, 5′-AUCUUUAUAGUGGGUAAUU-3′, and siRNA3, 5′-GUAUCUUUAUAGUGGGUAA-3′) was designed to target the back-spliced junction in the C190 by using the circular RNA Interactome database (http://circinteractome.nia.nih.gov, accessed on 16 February 2021). As shown in Figure 2A, a transwell migration assay was performed. The A549 cells with knockdown of C190 by siRNA2 and siRNA3 showed significantly decreased migration ability, compared with NC (* *p* < 0.05, ** *p* < 0.01). As shown in Figure 2B, a transwell invasion assay was performed after transfection of siRNA2 or SiRNA3 for 72 h. The A549 cells with knockdown of C190 by siRNA2 and siRNA3 showed significantly decreased invasion ability, compared with NC (*** *p* < 0.001). As shown in Figure 2C, a proliferation assay was performed. The A549 cells with knockdown of C190 by siRNA2 and siRNA3 showed significantly decreased proliferation ability, compared with NC (*p* = 0.0113, and 0.0142, respectively). As shown in Figure 2D, a wound-healing assay was performed. The A549 cells with knockdown of C190 by siRNA2 and siRNA3 showed a tendency toward decreased wound-healing ability, compared with the NC cells.

### 2.3. Overexpression of C190 Enhanced Tumor Growth In Vivo

**Figure 3:**
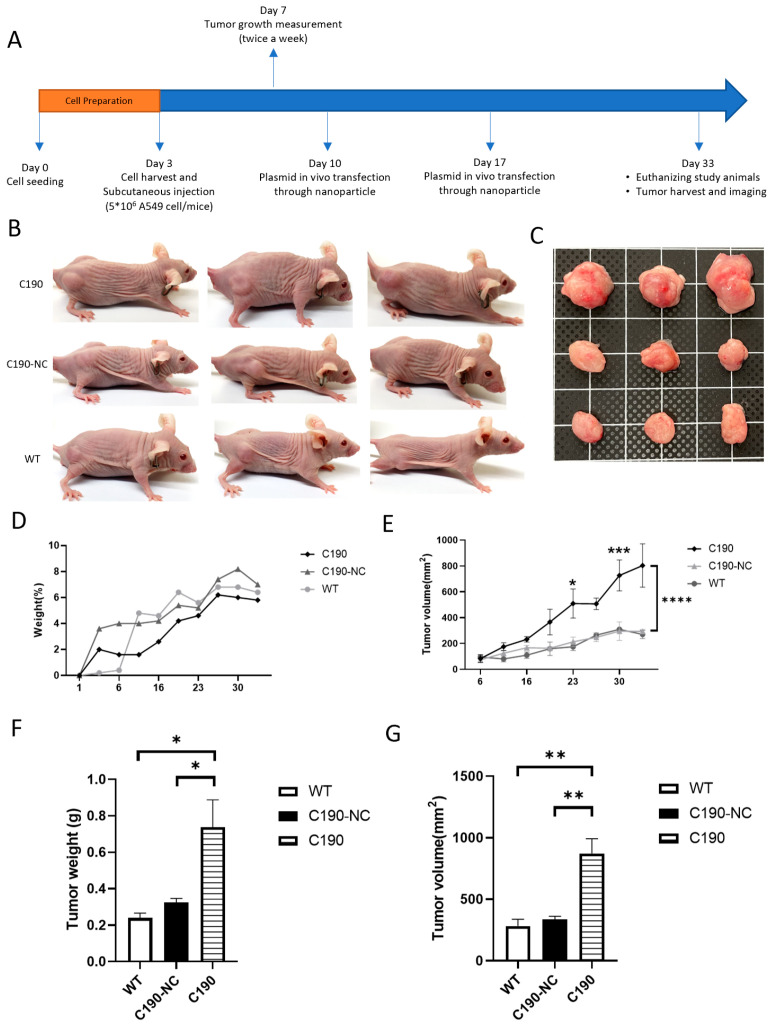
Overexpression of C190 enhances tumor growth: (**A**) Hsa_circ_0000190 overexpression xenograft experiment was performed for four weeks; (**B**) xenograft tumors were observed in the dorsolateral flank region of mice; (**C**) xenograft tumors were harvested from indicated groups; (**D**) the change in weight of mice in each group during the experiment until euthanasia was not significantly different; (**E**) the tumors derived from C190-overexpressing A549 cells showed significantly higher growth rate than those derived from the control groups (* *p* < 0.05, *** *p* < 0.001, **** *p* < 0.0001); (**F**) the tumor weight and (**G**) volume of C190-overexpressing tumors were significantly higher than those of the control group (** *p* < 0.01)(*n* = 5).

A549 cells were xenografted into immunocompromised mice, including the wild-type (WT) group, the C190-overexpression-negative control group (C190-NC), and the C190-overexpression group (C190). The tumor growth in the subcutaneous xenograft model was monitored in vivo for four weeks. After subcutaneous injection of pcDNA3.1(+) ZKSCAN1 MCS Exon Vector and pcDNA3.1(+) ZKSCAN1 MCS Exon Vector with hsa_circ_0000190 gene using nanoparticle transfection technology, the changes in tumor size and weight of mice were measured twice a week (Figure 3A). Xenograft tumors were located in the dorsolateral flank region of mice (Figure 3B). Xenograft tumors were harvested from indicated groups (Figure 3C). The change of mouse weight for each group showed no significant difference (Figure 3D). The tumors derived from C190-overexpressing A549 cells demonstrated a significantly higher growth rate than those derived from the empty vector-transfected control cells (Figure 3E). On the final day of the animal experiment, the tumor weight and volume of C190-overexpressing tumors were significantly higher than those of the control group (Figure 3F,G). 

### 2.4. Nanoparticle-Wrapped, siRNA-Mediated C190 Knockdown Inhibited Tumor Growth In Vivo

**Figure 4:**
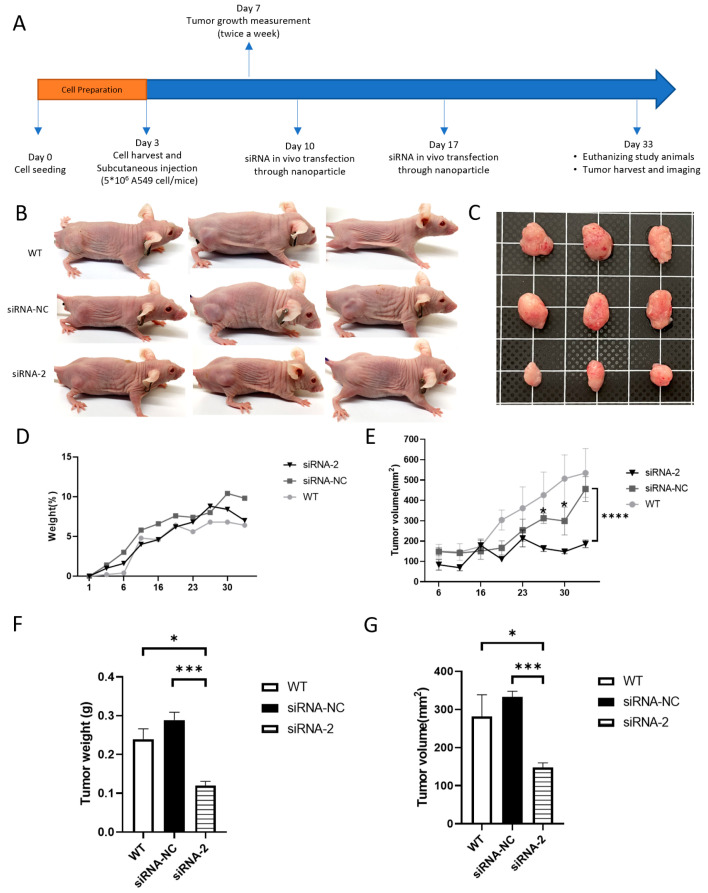
Nanoparticle-wrapped siRNA-mediated C190 knockdown inhibited tumor growth in vivo: (**A**) Hsa_circ_0000190 knockdown xenograft experiment was performed for four weeks; (**B**) xenograft tumors were observed in the dorsolateral flank region of mice; (**C**) xenograft tumors were harvested from indicated groups; (**D**) the change in weight of mice in each group during the experiment until euthanasia was not significantly different; (**E**) the tumors derived from A549 cells with nanoparticle-wrapped, siRNA-mediated C190 knockdown showed significantly lower growth rate than those derived from the control groups (* *p* < 0.05, **** *p* < 0.0001); (**F**) the tumor weight and (**G**) volume of C190 knockdown tumors were significantly lower than those of the control group (*** *p* < 0.001) (*n* = 5).

A549 cells were xenografted into immunocompromised mice, including the WT group, the non-targeting siRNA-negative control group (siRNA-NC), and the C190 siRNA group (siRNA-2). The tumor growth in the subcutaneous xenograft model was monitored in vivo for four weeks. After subcutaneous injection of non-targeting siRNA or siRNA2 using nanoparticle transfection technology, the changes in tumor size and weight in mice were measured twice a week (Figure 4A). Xenograft tumors were located in the dorsolateral flank region of mice (Figure 4B). Xenograft tumors were harvested from indicated groups (Figure 4C). Resected tumors showed various shapes, including flattened, nodular, and irregular shapes (Figure 3C and Figure 4C). The change in mouse weight for each group showed no significant difference (Figure 4D). The tumors derived from A549 cells with nanoparticle-wrapped, siRNA-mediated C190 knockdown demonstrated a significantly lower growth rate than the negative control groups (Figure 4E). On the final day of the animal experiment, the tumor weight and volume of C190 knockdown tumors were significantly lower than those of the negative control groups (Figure 4F,G). As shown in Figure 4G, the average tumor volume of the siRNA-NC group on the final day was numerically higher than that of the WT group. However, as evident in Figure 4E, the average tumor volume of the WT group on the final day was numerically higher than that of the siRNA-NC group. The reason for this difference is that the shapes of the tumors are quite irregular, so the measured volume of the subcutaneous tumors differed from that of the resected tumors. The irregular shapes of tumors could also result in the difference between the average tumor volume of the WT group in Figure 4E and that in Figure 3E. Nonetheless, the average tumor weight and volume of the WT groups in Figure 4F,G are similar to those in Figure 3F,G.

### 2.5. Interaction among hsa_circ_0000190, MicroRNA, and Various Immune Checkpoints

**Figure 5:**
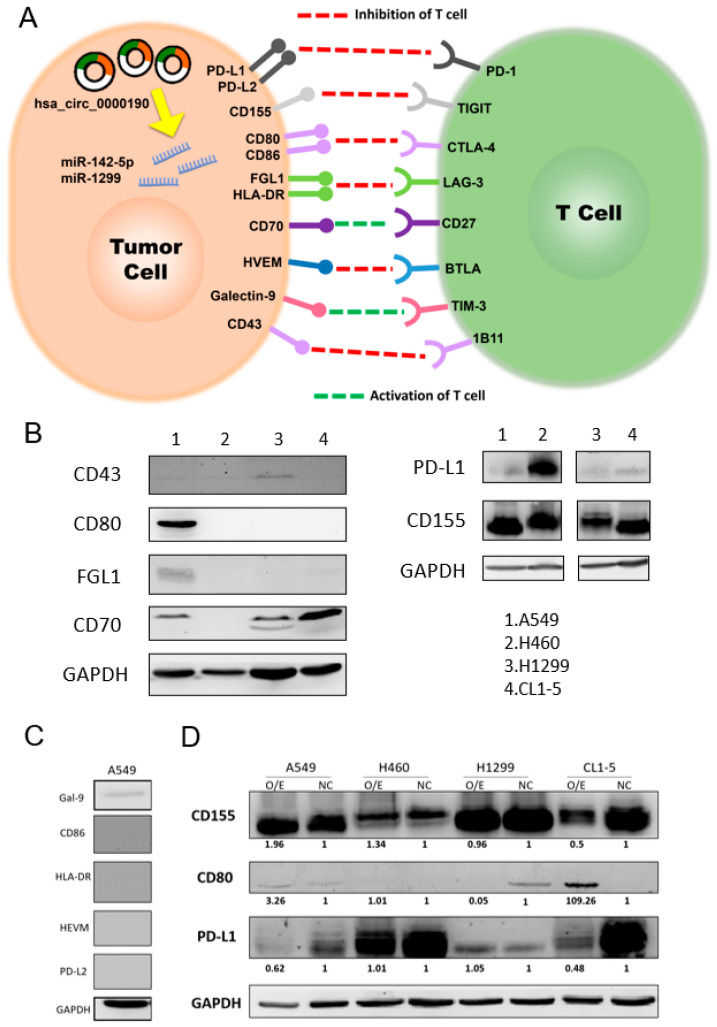
The relation between immune checkpoints and has_circ_0000190: (**A**) the potential influence of circRNA–miRNA–mRNA axis on the interaction of immune checkpoint receptors on the T cells and their respective ligands on the tumor cells; (**B**) the expression of different immune-checkpoint-related binding partners in different cell lines; (**C**) immune checkpoints that were not significantly detected in A549 cells; (**D**) the expression of immune checkpoints in cells line with overexpression of hsa_circ_0000190. All experiments were performed independently in triplicate.

The interaction among hsa_circ_0000190, microRNA, and various immune checkpoints were performed in this study. Based on the expression level of our NGS data in our previous study, we selected one most suppressed miRNA, miR1299 as a target of hsa_circ_0000190 [12], and another miRNA, miR-142-5p, which regulated antitumor immunity [24,25], for further miRNA analysis (Figure 5A). It has been reported that miR-142-5p is the downregulated miRNA target of C190 in NSCLC [26], and downregulation of miR-142-5p induces PD-L1 expression in tumor cells and regulates antitumor immunity [24,25].

### 2.6. PD-L1, CD155, CD80, FGL1, and CD70 Were Expressed in A549 Cells

The immune checkpoints include PD-1, TIGIT, CTLA-4, LAG-3, CD27, BTLA, TIM-3, and 1B11. Their related binding partners were PD-L1/PD-L2, CD155 (PVR), CD80/CD86, FGL1/HLA-DR, CD70, HVEM, galectin-9 (Gal-9), and CD43, respectively [27,28,29]. The expression of the different immune-checkpoint-related binding partners in various lung cancer cell lines is shown in Figure 3. Figure 5B shows that PD-L1, CD155, CD80, FGL1, and CD70 were expressed in A549 cells. Figure 5C shows that CD86, HVEM, PDL-2, HLA-DR were not detected in A549 cells. Only a weak expression of Gal-9 was found.

### 2.7. Overexpression of hsa_circ_0000190 Regulated Expression of Immune Checkpoints In Vitro

Figure 5D shows decreased expression of PD-L1 in A549 and CL1-5 cell lines with overexpression (O/E) of hsa_circ_0000190 (C190). Increased expression of CD80 in A549 and CL1-5 cell lines with overexpression of C190 was also found.

### 2.8. The Overexpression of hsa_circ_0000190 Promoted Expression of Specific Immune Checkpoint mRNAs in NSCLC Cells

**Figure 6:**
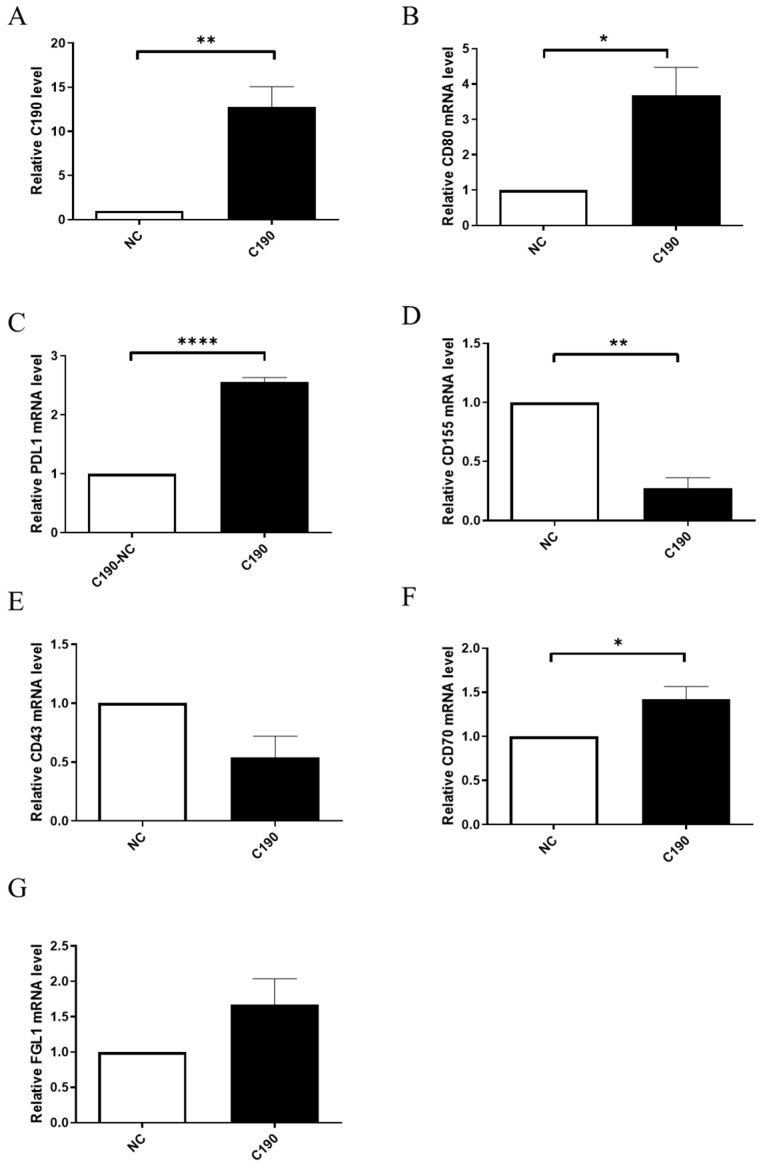
The overexpression of hsa_circ_0000190 promoted expression of specific immune checkpoint mRNAs in A549 cells: (**A**) the expression of C190 was significantly increased (** *p* < 0.01); (**B**,**C**,**F**) significantly increased mRNAs of CD80, PD-L1, and CD70 was found in A549 cells with overexpression of C190 (* *p* < 0.05, **** *p* < 0.0001); (**D**) significantly decreased mRNA of CD155 was observed; (**E**,**G**) no significantly different mRNA expression of CD43 and FGL1 was detected. All experiments were performed independently in triplicate.

To analyze the influence of C190 on immune-checkpoint mRNA in NSCLC by RT-qPCR, overexpression vectors targeting C190 were delivered to A549 cells. Figure 4 shows the analysis of immune-checkpoint mRNAs (CD80, PD-L1, CD155, CD-43, CD-70, and FGL1) by RT-qPCR. After transfection with the C190-overexpression vector in A549 cells, the expression of C190 was significantly increased (Figure 6A). Significantly increased mRNAs of CD80, PD-L1, and CD70 were found in A549 cells with overexpression of C190 (Figure 6B,C,F). Significantly decreased mRNA of CD155 was also found (Figure 6D).

### 2.9. The Knockdown of hsa_circ_0000190 by siRNA Impeded Expression of Specific Immune Checkpoint mRNAs in NSCLC Cells

**Figure 7:**
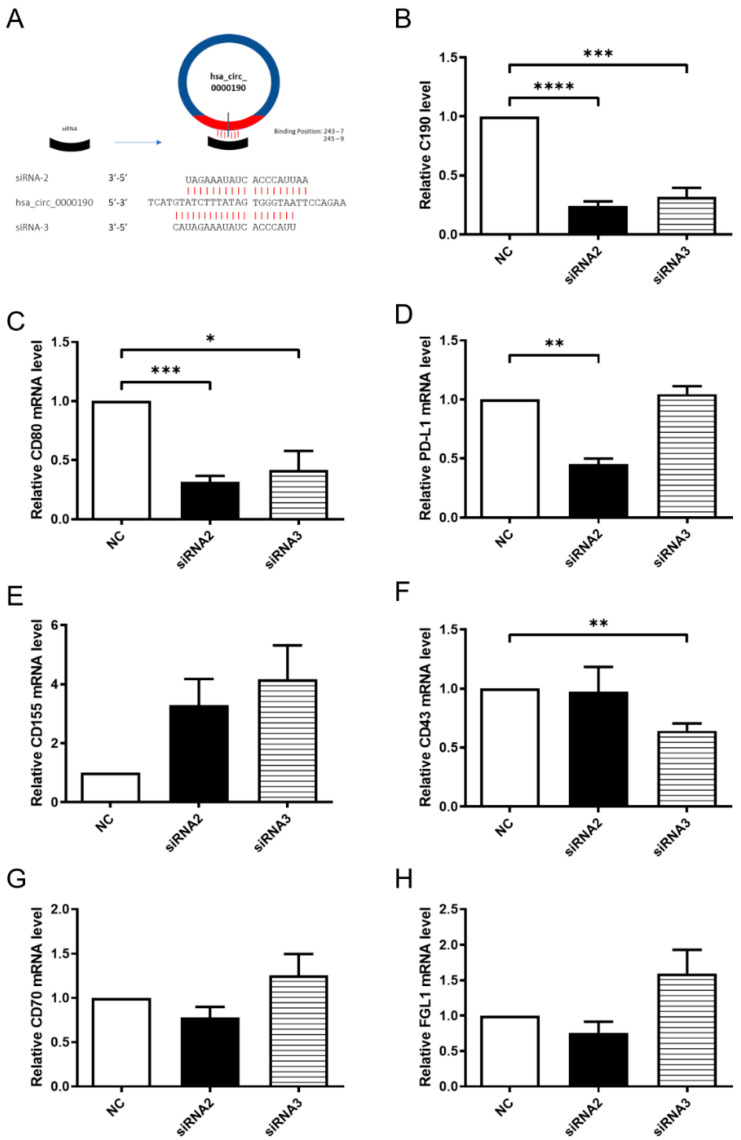
The knockdown of hsa_circ_0000190 (C190) by siRNA inhibited expression of specific immune checkpoint mRNAs in A549 cells: (**A**) siRNA-2 and siRNA-3 were designed to specifically target the back-splice junction site of C190; (**B**) significantly decreased expression of C190 after knockdown by siRNAs (*** *p* < 0.001, **** *p* < 0.0001); (**C**–**H**) significantly decreased mRNAs of CD80, PD-L1, CD43, CD70 and FGL, and CD43 were found in A549 cells with knockdown of C190 (* *p* < 0.05, ** *p* < 0.01). All experiments were performed independently in triplicate.

To analyze the influence of C190 on immune-checkpoint mRNAs in NSCLC by RT-qPCR, siRNA-targeting C190 was used for its expression knockdown. The specific siRNAs were designed to target the back-spliced junction in C190 (Figure 7A). After the knockdown of C190 by siRNA2 or siRNA3 in A549 cells, the expression of C190 was significantly decreased (Figure 7B). Further analysis of immune-checkpoint mRNA (CD80, PD-L1, CD155, CD43, CD70, and FGL1) was performed. Significantly decreased mRNAs of CD80, PD-L1, and CD43 were found in A549 cells with knockdown of C190 (Figure 7C,D,F). 

### 2.10. Overexpression of hsa_circ_0000190 Increased the Expression and Secretion of Soluble PD-L1 in NSCLC Cell Lines and May Be Associated with Decreased Efficacy of Anti-PD-1 Antibody

**Figure 8:**
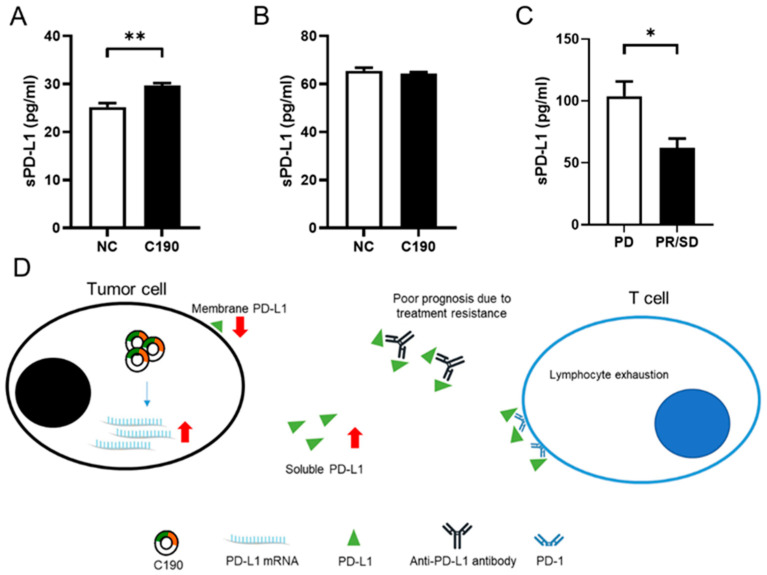
Overexpression of hsa_circ_0000190 increased soluble PD-L1 production in NSCLC cells and may be associated with decreased efficacy of anti-PD-1 antibody: (**A**) significantly increased sPD-L1 was detected in the conditioned medium of A549 cells with C190 overexpression, compared with the negative control (** *p* < 0.01); (**B**) C190 overexpression did not increase sPD-L1 in the conditioned medium of H460 cells; (**C**) Patients with progressive disease (PD) after immunotherapy had higher expression of sPD-L1, compared with those with partial regression (PR) or stable disease (SD) (* *p* < 0.05); (**D**) tumor cells with C190 expression may hinder the efficacy of anti-PD-L1 antibody and T-cell activation through increased sPD-L1, leading to treatment resistance and poor prognosis. All experiments were performed independently in triplicate.

Our findings showed that significantly increased mRNAs of PD-L1 were found in A549 cells with overexpression of C190, and significantly decreased mRNAs of PD-L1 were found in A549 cells with knockdown of C190. However, the Western blot analysis showed decreased expression of PD-L1 in A549 cells with overexpression of C190. Therefore, the PD-L1 expression and secretion to the culture medium of A549 and H460 cells were evaluated. As evident in Figure 7, the expression of soluble PD-L1 (sPD-L1) in the conditioned medium of A549 and H460 cells was evaluated by ELISA. After transfection of C190 overexpression vector for 48 h, culture supernatant of cells was collected, and significantly increased sPD-L1 was detected in the conditioned medium of A549 cells (Figure 8A). However, C190 overexpression did not increase sPD-L1 in the conditioned medium of H460 cells (Figure 8B). Accumulating data have revealed that elevated levels of plasma sPD-L1 are associated with decreased efficacy of anti-PD-1 monoclonal antibody (mAb) and worst outcomes [30,31]. The sPD-L1 in the plasma from patients (*n* = 30) with advanced NSCLC before receiving immunotherapy was evaluated. Patients with progressive disease (PD) after immunotherapy had higher expression of sPD-L1, compared with those with partial regression (PR) or stable disease (SD) (Figure 8C). According to our current and previous findings [12], tumor cells with C190 expression may hinder the efficacy of anti-PD-L1 antibody and T-cell activation through increased sPD-L1, leading to treatment resistance and poor prognosis (Figure 8D).

## 3. Discussion

Cumulative evidence suggested the involvement of circular RNAs in the pathogenesis and progression of NSCLC, such as hsa_circ_0000190, circular RNA circ_0000284, circ-CPA4, etc. [12,20,22]. Our previous finding showed that upregulated C190 levels correlated with poor response to systemic therapy and immunotherapy. The current investigation illuminated that c190 contributed to the tumorigenesis and immune escape of NSCLC by promoting sPD-L1 expression, implicating a novel insight into the pathological mechanism of PD-L1-dependent immune evasion in NSCLC.

Previously, C190 was found to be upregulated and was associated with later-stage, more distant metastatic organs metastasis, poor survival, and poor response to systemic and immunotherapy [12,32]. In accordance with the reported data, this research revealed that the overexpression of C190 promoted tumorigenesis of NSCLC, including migration, invasion, and wound healing. Conversely, knockdown of C190 brought about the suppression of NSCLC tumorigenesis. The nanoparticle-based siRNA delivery system targeting C190 in this study could significantly decrease tumor growth and may be a potential tool for cancer therapy. Multiple early phase clinical trials regarding nanoparticle-based siRNA for cancer treatment are ongoing [33]. Therefore, these results implied that C190 might serve as a prognostic indicator of NSCLC, and the inhibition of C190 might be adopted as a therapeutic strategy to block the progression of NSCLC. Downregulation of C190 has been reported in multiple myeloma and gastric cancer, whereas C190 displays upregulation in lung cancer [12,34,35]. These findings highlight the cell and tissue specificity of circRNAs, which may affect different biological processes, showing that their dysregulation and potential function should be elucidated in various types of cancers [36].

Multiple studies have demonstrated the role of circRNAs in regulating the expression of PD-L1 through the circRNA–miRNA–mRNA axis. CircCHST15 facilitated the PD-L1-mediated immune escape of lung cancer cells by inhibiting miR-155-5p and miR-194-5p [19]. The circ-CPA4–let-7 miRNA–PD-L1 axis regulated the growth of NSCLC cells and inhibited cytotoxic T cells in TIME [20]. Hsa-circRNA-002178 in lung cancer cells promoted the expression of PD-L1 through sponging miR-34 to cause T-cell exhaustion [21]. Circ_0000284 promoted upregulation of PD-L1 through inhibiting miR-377 in NSCLC [22]. Accumulating evidence suggested that circRNAs play an important role in TIME and in regulating immune-checkpoint genes, to affect the therapeutic efficacy of immune-checkpoint inhibitors. Further investigation regarding the regulatory mechanism of circRNAs in antitumor immunity and the efficacy of immunotherapy will benefit the therapeutic strategies of lung cancer [23]. In our study, upregulated plasma hsa_circ_0000190 level has been reported to correlate with poor response to immunotherapy and the expression of PD-L1 [12], and a potential regulatory mechanism between C190 and PD-L1 was investigated in this research. Specifically, by evaluating the cell lines and clinical specimens, PD-L1 mRNA could be elevated by overexpression of C190, and sPD-L1 expression was increased subsequently. However, the expression levels of PD-L1 in the tumor cells was decreased, suggesting that C190-related expression of PD-L1 was predominantly in the sPD-L1 pattern but not in the membrane PD-L1 pattern. In addition, poor response to immunotherapy in patients with higher expression of sPD-L1 was also found. A previous report also showed that NSCLC cells inhibited cytotoxic T cells through secreted PD-L1-dependent mechanisms [20]. Collectively, we uncovered the regulatory mechanisms of C190, PD-L1, and sPD-L1 in NSCLC cells and found that C190 overexpression promoted sPD-L1 expression without simultaneously elevated membrane PD-L1 expression. NSCLC with C190 expression may inhibit the efficacy of anti-PD-L1 antibody and T-cell activation through increased sPD-L1, leading to immunotherapy resistance and poor outcome. Immunotherapy with ant-PD-L1 mAb plus inhibition of C190 by siRNA might be further investigated as a therapeutic strategy to impede the progression of NSCLC.

Currently, immunotherapy resistance in NSCLC remarkably limited their therapeutic efficacy [37], and the identification of novel therapeutic strategies to improve the effect of immunotherapy remains critical. It has been reported that PD-L1-containing exosomes from tumor cells resulted in drug resistance [38]. A circular RNA, circ-CPA4, was found to positively regulate exosomal PD-L1, and NSCLC with circ-CPA4 inhibition reactivated cytotoxic T cells in vivo [20]. In our study, C190 upregulated sPD-L1, which was associated with poor response to the immunotherapy, suggesting that inhibition of C190 may potentially facilitate the efficacy of anti-PD-L1 antibody by decreasing the production of sPD-L1. Impaired immune surveillance with subsequent immune evasion was found in NSCLC [39,40]. We found that C190 expression regulated immune-checkpoint-related binding partners including CD80 and PD-L1, which may have an influence on the immune evasion in the tumor microenvironment. These findings suggested that circRNAs played important roles in the resistance to an immune-checkpoint inhibitor, providing potential therapeutic targets to improve treatment efficacy [41]. Further studies are necessary to elucidate this strategy.

## 4. Materials and Methods

### 4.1. Cell Culture

Human lung cancer cell lines were obtained from the American Type Culture Collection (ATCC) and tested positive for human origin [12,42]. The human lung cancer cells (CL1-0 to CL1-5, A549, H460, and H1299) were cultured in DMEM with 10% fetal bovine serum, 100 units/mL penicillin, and 100 μg/mL streptomycin under standard culture conditions (37 °C, 95% humidified air and 5% CO_2_). Subcultures were performed with trypsin-EDTA. Media were refreshed every two days or three days.

### 4.2. Cell and Plasmid Preparation for Overexpressing Circular RNA

All plasmids were amplified by DH5-alpha competent *E. coli* cells. For preparation, 2 µL (100 ng/µL) of Plasmid DNA was added to 50 µL DH5-alpha competent cells and placed on ice for 25 min. Heat shock was performed at 42 °C for 45 s. Then, the specimens were placed on ice for 2 min, and 250 µL room temperature LB medium was pipetted into the mixture. After incubation of the specimens at 37 °C for 30 min, 50–100 µL of them was spread onto a selection plate and incubated overnight at 37 °C. A single clone was selected into a 40 mL LB medium that contained ampicillin (100 µg/mL) and incubated overnight at 37 °C. Overnight cultures were extracted by QIAGEN Plasmid Maxi Kit (QIAGEN, catalog No. 12163, Hilden, Germany) and measured by microvolume spectroscopy (Nano Photometer^®^ N60/N50, IMPLEN, Westlake Village, CA, USA). The plasmid was diluted to a final concentration of 1 ug/ul for transfection. The TransIT^®^-LT1 Transfection Reagent (Mirus, MIR2300, Madison, WI, USA) was used for the DNA transfection according to the manufacturer’s instructions. Plasmids used for the circular RNA overexpression consist of pcDNA3.1(+) ZKSCAN1 MCS Exon Vector (Addgene, plasmid #69901, Cambridge, MA, USA) and pcDNA3.1(+) ZKSCAN1 MCS Exon Vector with hsa_circ_0000190 gene.

### 4.3. Transfection with Small-Interfering RNA (siRNA)

Cells (2 × 10^5^/mL) were seeded in 6-well plates. After 24 h of incubation, cells were transfected using Lipofectamine™ 2000 Transfection Reagent (Invitrogen), according to the manufacturer’s instructions. Hsa_circ_0000190 siRNAs were synthesized by MDBio (Taipei, Taiwan). Transfection efficiency in the cell lines was confirmed by RT-qPCR. Three days after transfection, cells were treated with the indicated reagent for further experiments.

### 4.4. Cell Proliferation Assay

Cell proliferation was measured by using Cell Counting Kit-8 (CK04-01, Dojindo, Kumamoto, Japan) according to the manufacturer’s instructions. Cell suspensions were plated to a density of 5000 cells/100 μL in 96-well culture plates after 24 h of further incubation, 10 μL of CCK-8 solution was added to each well. The plates were read in the ELISA plate reader at 450 nm with a reference wavelength of 650 nm. All experiments were performed independently in triplicate.

### 4.5. Migration and Invasion

Transwell assays were performed by using Transwell plates (Falcon, Franklin Lakes, NJ, USA). The cells were allowed to migrate through an 8.0 µm transparent PET membrane (Falcon, Franklin Lakes, NJ, USA), and the matrigel invasion Chamber (BD, Bedford, MA, USA) was used for invasion analysis. The expression of hsa_circ_0000190 in lung cancer cells was transiently overexpressed or knocked down for 72 h. In total, 1–2 × 10^5^ cells with 1% FBS medium were added in each insert, and 10% FBS medium was placed in each lower chamber. After 24 h of incubation at 37 °C, inserts were taken out and counted under an inverted fluorescent microscope [18]. The average number of cells and images from the microscopic fields of three experiments were recorded. Each image was taken from the center of the well.

### 4.6. Wound-Healing Assay

Cells (5 × 10^5^/mL) were seeded in 6-well plates and cultured for 24 h. A pipette tip was used to make a straight scratch after 24 h. Then, the bottom of the plate was marked, and the image was recorded. After image recording, the expression of hsa_circ_0000190 in lung cancer cells was transiently overexpressed or knocked down. Images were recorded at 0 h, 24 h, and 72 h. Finally, the Image J software (version 1.8.0, National Institute of Health, Bethesda, MD, USA) was used to calculate the percentage of wound closure.

### 4.7. Animal Experiments

All animal experiments were approved by the Institutional Animal Care and Use Committee of Taipei Veterans General Hospital. Male BALB/c nude mice (7–8 weeks old) (National Laboratory Animal Center, Taipei, Taiwan) were divided into different groups, including the wild-type (WT) group (*n* = 5), the C190-overexpression-negative control group (*n* = 5), the C190-overexpression group (*n* = 5), the siRNA-negative control group (*n* = 5), and the C190 siRNA group (*n* = 5). The A549 cells were harvested and subjected to subcutaneous implantation into the dorsolateral flank region of the mice. The wild-type A549 cells were inoculated into the WT group. By using NANOPARTICLE-based In Vivo Transfection Reagent (Altogen, Catalog #5031, Las Vegas, NV, USA) to improve cell penetration and prolong half-life, the pcDNA3.1(+) ZKSCAN1 MCS Exon Vector and pcDNA3.1(+) ZKSCAN1 MCS Exon Vector with hsa_circ_0000190 gene were wrapped in the nanoparticles and subcutaneously injected into the C190-overexpression-negative control group and the C190-overexpression group, respectively. Then, 50 μL nanoparticles coupled with 100 μg scrambled siRNA (5′-UUCUCCGAACGUGUCACGU-3′) or siRNAs targeting C190 were subcutaneously injected into the siRNA-negative control group or the C190 siRNA group, respectively. The tumor size was measured twice a week using a caliper. The average tumor volume was calculated by the following equation: V = ½ (A × B^2^) (A, long diameter; B, short diameter) [32]. The mice were euthanized after 28 days of A549 cell inoculation. Then, xenograft tumors were removed and imaged. The weight and size of the tumors were measured, and the tumor tissues were collected for analysis.

### 4.8. Western Blotting Analysis

Total protein of human lung cancer cells was extracted with RIPA lysis and extraction buffer (Thermo Fisher, Catalog #89900, Waltham, MA, USA) according to the manufacturer’s instructions. An equal amount of the total protein was measured by Protein Assay Dye Reagent Concentrate (Bio-Rad, Catalog #5000001, Hercules, CA, USA). For evaluation of the association between immune checkpoints and hsa_circ_0000190, relevant ligands were selected, such as CD80/CD86, which interacted with CTLA-4 and CD28 for T-cell inhibition or activation, respectively. The other immune checkpoints included TIM-3, BTLA, TIGIT, LAG-3, and PD-1, and the related binding partners were galectin-9, HVEM, PVR (CD155), HLA-DR, and PD-L1, respectively [13,14,15,16,17]. 

An equal amount of the total cell lysates was subjected to Western blotting analysis by using anti-PD-L1 (Abcam, ab205921, Cambridge, MA, USA), anti-CD80 (Abcam, ab224797, USA), anti-CD86 (Abcam, ab269587, USA), anti-HVEM (Abcam, ab62462, USA), anti-HLA-DR (Abcam, ab92511, USA), anti-galectin-9 (Abcam, ab123712, USA), anti-CD155 (Abcam, ab229553, USA), anti-GAPDH (Taiclone, tcba13660, Taipei, Taiwan), anti-CD43 (Abcam, ab233969, USA), anti-CD70 (Abcam, ab175389, USA), anti-FGL1 (Abcam, ab197357, USA), and anti-PD-L2 antibody (Abcam, ab256386, USA) as primary antibodies. Goat anti-rabbit horseradish peroxidase (HRP) (Abcam, ab6712, USA) and goat anti-mouse HRP (Abcam, ab97023, USA) were used as secondary antibodies. Immunoreactive bands were visualized by Western Lightning Plus-ECL, Enhanced Chemiluminescence Substrate (PerkinElmer, NEL103001EA, Waltham, MA, USA).

### 4.9. RNA Extraction and RT-qPCR

Total RNA was extracted by RNeasy Mini Kit (QIAGEN, Hilden, Germany). The amount of the total RNA was measured by microvolume spectroscopy (IMPLEN, USANanoPhotometer^®^ N60/N50). For the RT-PCR, 5 ug of total RNA for the cDNA synthesis, random hexamer primer (Thermo Fisher, SO142, Waltham, MA, USA), and reverse transcriptase (Thermo Fisher, Waltham, MA, USA) were used according to the manufacturer’s instructions. PCRs were performed by using the SYBR Green method in an ABI 7000 sequence detection system (Applied Biosystems). The primer sequences are listed in Supplementary Materials Table S1.

### 4.10. Patient Population

Whole blood samples will be collected from patients with advanced lung cancer receiving treatment, including PD-l/L1 blocking antibodies. The medical records are reviewed to evaluate the clinical characteristics. Blood specimens will be collected before receiving immunotherapy. 

### 4.11. Enzyme-Linked Immunosorbent Assay

The sPD-L1 level was measured using the Human PD-L1SimpleStep ELISA^®^ Kit (Abcam, ab214565) according to the manufacturer’s instructions. All the samples were tested in duplicate.

### 4.12. Lung Cancer Treatment Efficacy Evaluation

The baseline evaluation of lung cancer was performed within 3 weeks prior to immunotherapy treatment. The chest computed tomography scan was performed within 3 weeks before starting treatment, and then, every 3 months thereafter, or when confirmation of treatment response or disease progression was needed. Treatment response assessment was analyzed according to the Response Evaluation Criteria in Solid Tumors (RECIST) group criteria (version 1.1). Progression-free survival (PFS) was calculated from the date of initiating treatment to the earliest sign of disease progression, as determined by the RECIST criteria, or death from any cause. If disease progression had not occurred at the time of the last follow-up visit, PFS was considered to have been censored at that time. Overall survival was measured from the date of initiating treatment until the date of death or last follow-up.

### 4.13. Statistical Analyses

The quantifiable data of the results are expressed as mean ± standard error of the mean (SEM). Differences between the groups were analyzed using one-way ANOVA, followed by Student’s *t*-test. When comparing response rate and immune biomarkers, the Mann–Whitney test was used for non-parametric data, and Pearson’s χ^2^ test was used for parametric data. The Statistical Package for the Social Sciences (SPSS) 20.0 software (SPSS, Chicago, IL, USA) and PRISM (GraphPad Software Inc., San Diego, CA, USA) were used for statistical analysis. All *p*-values were two sided, and a value of <0.05 was considered statistically significant.

## 5. Conclusions

In summary, hsa_circ_0000190 was shown to increase the PD-L1 mRNA-mediated sPD-L1 expression, consequently interfering with the efficacy of anti-PD-L1 antibody and T-cell activation, which may result in immunotherapy resistance and poor outcomes. Our results unraveled that hsa_circ_0000190 facilitated the tumorigenesis and immune evasion of NSCLC by upregulating sPD-L1 expression, potentially developing a different aspect in elucidating the molecular immunopathogenesis of NSCLC. Hsa_circ_0000190 upregulation can be an effective indicator for the progression of NSCLC, and hsa_circ_0000190 downregulation may possess a potential therapeutic value for the treatment of NSCLC in combination with immunotherapy.

## Data Availability

The data supporting the reported results of this study are included in the article.

## Supplementary Materials

The following are available online at https://www.mdpi.com/article/10.3390/ijms23010064/s1.

## References

[1] Ettinger D.S., Akerley W., Borghaei H., Chang A.C., Cheney R.T., Chirieac L.R., D’Amico T.A., Demmy T.L., Ganti A.K., Govindan R. (2012). Non-small cell lung cancer. J. Natl. Compr. Cancer Netw..

[2] Tan C.-S., Gilligan D., Pacey S. (2015). Treatment approaches for EGFR-inhibitor-resistant patients with non-small-cell lung cancer. Lancet Oncol..

[3] Hanna N., Johnson D., Temin S., Baker S., Brahmer J., Ellis P.M., Giaccone G., Hesketh P.J., Jaiyesimi I., Leighl N.B. (2017). Systemic Therapy for Stage IV Non-Small-Cell Lung Cancer: American Society of Clinical Oncology Clinical Practice Guideline Update. J. Clin. Oncol..

[4] Chen Y.M. (2013). Update of epidermal growth factor receptor-tyrosine kinase inhibitors in non-small-cell lung cancer. J. Chin. Med. Assoc..

[5] Chen Y.M., Whang-Peng J., Chen C.M. (2011). First-line Systemic Therapy for Metastatic Non-small-cell Lung Cancer—A Review. J. Exp. Clin. Med..

[6] Postow M.A., Callahan M.K., Wolchok J.D. (2015). Immune Checkpoint Blockade in Cancer Therapy. J. Clin. Oncol..

[7] Ribas A. (2012). Tumor immunotherapy directed at PD-1. N. Engl. J. Med..

[8] Luo Y.H., Ho H.L., Tsai C.M., Shih J.F., Chiu C.H., Lai S.L., Lee Y.C., Perng R.P., Whang-Peng J., Chou T.Y. (2015). The association between tumor epidermal growth factor receptor (EGFR) mutation and multiple primary malignancies in patients with adenocarcinoma of the lungs. Am. J. Clin. Oncol..

[9] Kramer M.C., Liang D., Tatomer D.C., Gold B., March Z.M., Cherry S., Wilusz J.E. (2015). Combinatorial control of Drosophila circular RNA expression by intronic repeats, hnRNPs, and SR proteins. Genes Dev..

[10] Su M., Xiao Y., Ma J., Tang Y., Tian B., Zhang Y., Li X., Wu Z., Yang D., Zhou Y. (2019). Circular RNAs in Cancer: Emerging functions in hallmarks, stemness, resistance and roles as potential biomarkers. Mol. Cancer.

[11] Zong L., Sun Q., Zhang H., Chen Z., Deng Y., Li D., Zhang L. (2018). Increased expression of circRNA_102231 in lung cancer and its clinical significance. Biomed. Pharmacother..

[12] Luo Y.H., Yang Y.P., Chien C.S., Yarmishyn A.A., Ishola A.A., Chien Y., Chen Y.M., Huang T.W., Lee K.Y., Huang W.C. (2020). Plasma Level of Circular RNA hsa_circ_0000190 Correlates with Tumor Progression and Poor Treatment Response in Advanced Lung Cancers. Cancers.

[13] Ge J., Wang J., Xiong F., Jiang X., Zhu K., Wang Y., Mo Y., Gong Z., Zhang S., He Y. (2021). Epstein-Barr Virus-Encoded Circular RNA CircBART2.2 Promotes Immune Escape of Nasopharyngeal Carcinoma by Regulating PD-L1. Cancer Res..

[14] Xu Y.J., Zhao J.M., Gao C., Ni X.F., Wang W., Hu W.W., Wu C.P. (2021). Hsa_circ_0136666 activates Treg-mediated immune escape of colorectal cancer via miR-497/PD-L1 pathway. Cell Signal.

[15] Jiang Z., Hou Z., Liu W., Yu Z., Liang Z., Chen S. (2021). Circ-Keratin 6c Promotes Malignant Progression and Immune Evasion of Colorectal Cancer through microRNA-485-3p/Programmed Cell Death Receptor Ligand 1 Axis. J. Pharmacol. Exp. Ther..

[16] Tanaka E., Miyakawa Y., Kishikawa T., Seimiya T., Iwata T., Funato K., Odawara N., Sekiba K., Yamagami M., Suzuki T. (2019). Expression of circular RNA CDR1AS in colon cancer cells increases cell surface PDL1 protein levels. Oncol. Rep..

[17] Gao C., Xu Y.J., Qi L., Bao Y.F., Zhang L., Zheng L. (2021). CircRNA VIM silence synergizes with sevoflurane to inhibit immune escape and multiple oncogenic activities of esophageal cancer by simultaneously regulating miR-124/PD-L1 axis. Cell Biol. Toxicol..

[18] Xu G., Zhang P., Liang H., Xu Y., Shen J., Wang W., Li M., Huang J., Ni C., Zhang X. (2021). Circular RNA hsa_circ_0003288 induces EMT and invasion by regulating hsa_circ_0003288/miR-145/PD-L1 axis in hepatocellular carcinoma. Cancer Cell Int..

[19] Yang J., Jia Y., Wang B., Yang S., Du K., Luo Y., Li Y., Zhu B. (2021). Circular RNA CHST15 Sponges miR-155-5p and miR-194-5p to Promote the Immune Escape of Lung Cancer Cells Mediated by PD-L1. Front. Oncol..

[20] Hong W., Xue M., Jiang J., Zhang Y., Gao X. (2020). Circular RNA circ-CPA4/ let-7 miRNA/PD-L1 axis regulates cell growth, stemness, drug resistance and immune evasion in non-small cell lung cancer (NSCLC). J. Exp. Clin. Cancer Res..

[21] Wang J., Zhao X., Wang Y., Ren F., Sun D., Yan Y., Kong X., Bu J., Liu M., Xu S. (2020). circRNA-002178 act as a ceRNA to promote PDL1/PD1 expression in lung adenocarcinoma. Cell Death Dis..

[22] Li L., Zhang Q., Lian K. (2020). Circular RNA circ_0000284 plays an oncogenic role in the progression of non-small cell lung cancer through the miR-377-3p-mediated PD-L1 promotion. Cancer Cell Int..

[23] Fang Z., Jiang C., Li S. (2020). The Potential Regulatory Roles of Circular RNAs in Tumor Immunology and Immunotherapy. Front. Immunol..

[24] Wan J., Ling X., Peng B., Ding G. (2018). MiR-142-5p regulates CD4+ T cells in human non-small cell lung cancer through PD-L1 expression via the PTEN pathway. Oncol. Rep..

[25] Jia L., Xi Q., Wang H., Zhang Z., Liu H., Cheng Y., Guo X., Zhang J., Zhang Q., Zhang L. (2017). MiR-142-5p regulates tumor cell PD-L1 expression and enhances anti-tumor immunity. Biochem. Biophys. Res. Commun..

[26] Wang Z., Liu Z., Fang X., Yang H. (2017). MiR-142-5p Suppresses Tumorigenesis by Targeting PIK3CA in Non-Small Cell Lung Cancer. Cell Physiol. Biochem..

[27] Wei S.C., Duffy C.R., Allison J.P. (2018). Fundamental Mechanisms of Immune Checkpoint Blockade Therapy. Cancer Discov..

[28] Torphy R.J., Schulick R.D., Zhu Y. (2017). Newly Emerging Immune Checkpoints: Promises for Future Cancer Therapy. Int. J. Mol. Sci..

[29] Batdorf B.H., Kroft S.H., Hosking P.R., Harrington A.M., Mackinnon A.C., Olteanu H. (2017). Evaluation of CD43 expression in non-hematopoietic malignancies. Ann. Diagn. Pathol..

[30] Khan M., Zhao Z., Arooj S., Fu Y., Liao G. (2020). Soluble PD-1: Predictive, Prognostic, and Therapeutic Value for Cancer Immunotherapy. Front. Immunol..

[31] Orme J.J., Enninga E.A.L., Lucien-Matteoni F., Dale H., Burgstaler E., Harrington S.M., Ball M.K., Mansfield A.S., Park S.S., Block M.S. (2020). Therapeutic plasma exchange clears circulating soluble PD-L1 and PD-L1-positive extracellular vesicles. J. Immunother. Cancer.

[32] Ishola A.A., Chien C.S., Yang Y.P., Chien Y., Yarmishyn A.A., Tsai P.H., Chen J.C., Hsu P.K., Luo Y.H., Chen Y.M. (2021). Oncogenic circRNA hsa_circ_0000190 modulates EGFR/ERK pathway in promoting NSCLC. Cancer Res..

[33] Hattab D., Gazzali A.M., Bakhtiar A. (2021). Clinical Advances of siRNA-Based Nanotherapeutics for Cancer Treatment. Pharmaceutics.

[34] Feng Y., Zhang L., Wu J., Khadka B., Fang Z., Gu J., Tang B., Xiao R., Pan G., Liu J. (2019). CircRNA circ_0000190 inhibits the progression of multiple myeloma through modulating miR-767-5p/MAPK4 pathway. J. Exp. Clin. Cancer Res..

[35] Chen S., Li T., Zhao Q., Xiao B., Guo J. (2017). Using circular RNA hsa_circ_0000190 as a new biomarker in the diagnosis of gastric cancer. Clin. Chim. Acta.

[36] Carrasco-Leon A., Amundarain A., Gomez-Echarte N., Prosper F., Agirre X. (2021). The Role of lncRNAs in the Pathobiology and Clinical Behavior of Multiple Myeloma. Cancers.

[37] Wang F., Wang S., Zhou Q. (2020). The Resistance Mechanisms of Lung Cancer Immunotherapy. Front. Oncol..

[38] Gao L., Guo Q., Li X., Yang X., Ni H., Wang T., Zhao Q., Liu H., Xing Y., Xi T. (2019). MiR-873/PD-L1 axis regulates the stemness of breast cancer cells. EBioMedicine.

[39] Lin A., Wei T., Meng H., Luo P., Zhang J. (2019). Role of the dynamic tumor microenvironment in controversies regarding immune checkpoint inhibitors for the treatment of non-small cell lung cancer (NSCLC) with EGFR mutations. Mol. Cancer.

[40] Santaniello A., Napolitano F., Servetto A., De Placido P., Silvestris N., Bianco C., Formisano L., Bianco R. (2019). Tumour Microenvironment and Immune Evasion in EGFR Addicted NSCLC: Hurdles and Possibilities. Cancers.

[41] Chen H.H., Zhang T.N., Wu Q.J., Huang X.M., Zhao Y.H. (2021). Circular RNAs in Lung Cancer: Recent Advances and Future Perspectives. Front. Oncol..

[42] Chu Y.W., Yang P.C., Yang S.C., Shyu Y.C., Hendrix M.J., Wu R., Wu C.W. (1997). Selection of invasive and metastatic subpopulations from a human lung adenocarcinoma cell line. Am. J. Respir. Cell Mol. Biol..

