# Supplementary material for: Circular RNA hsa_circ_0000190 Facilitates the Tumorigenesis and Immune Evasion by Upregulating the Expression of Soluble PD-L1 in Non-Small-Cell Lung Cancer
Source: Int J Mol Sci. 2021 Dec 22;23(1):64. doi: 10.3390/ijms23010064 (PMC8744551; doi:10.3390/ijms23010064)
Supplement: Supplementary file 1 [file ijms-23-00064-s001.zip › ijms-1486545-supplementary.pdf]

Supplementary materials

Supplementary Materials Table S1: List of nucleic acid sequences for qPCR Primers

qPCR Primer		
**Gene name**	**Forward sequence**	**Reverse sequence**
Hsa_circ_0000190	GGCAGCTGAAGTCACACATGAA	CCAGTGCAATGACATGAGCAGT
GAPDH	GTCTCCTCTGACTTCAACAGCG	ACCACCCTGTTGCTGTAGCCAA
PD-Ll	TGCCGACTACAAGCGAATTACTG	CTGCTTGTCCAGATGACTTCGG
CD80	CTCTTGGTGCTGGCTGGTCTTT	GCCAGTAGATGCGAGTTTGTGC
CD43	GCAACCAGTCATCCTGCTGTTC	CTGGTCCTACTGGAGGTTTCTG
CD155	CACTGTCACCAGCCTCTGGATA	TCATAGCCAGAGATGGATACCTC
CD70	TTCGCACAGGCTCAGCAGCAG	TTGTCCAGCTCTGGTCCATGCA
FGL1	GAAGATCAGTCTGGCTGGTGGT	TGCCAGGTGTACCAGACAATCC
